# Hyperbaric oxygen therapy as adjuvant therapy for odontogenic necrotizing myositis: A case report

**DOI:** 10.1002/ccr3.4726

**Published:** 2021-08-30

**Authors:** Andrea N. Cracchiolo, Daniela Maria Palma, Marco Palmeri, Diego Tantillo, Rosalia Lo Bue, Andrea Braconi, Claudio Caramanna, Luigi Solazzo, Fabio Genco, Paola Mirto

**Affiliations:** ^1^ UOC Emergenza Urgenza 118 Servizio di Medicina Iperbarica ARNAS Ospedale Civico Di Cristina Benfratelli Palermo Italy; ^2^ UOS Terapia Intensiva e Trauma Center ARNAS Ospedale Civico Di Cristina Benfratelli Palermo Italy; ^3^ UOC Chirurgia Maxillo‐Faciale ARNAS Ospedale Civico Di Cristina Benfratelli Palermo Italy; ^4^ Basingstoke and North Hampshire Hospital Hampshire UK

**Keywords:** adjuvant therapy, hyperbaric oxygen therapy, necrotizing myositis, odontogenic infections, soft tissue infections

## Abstract

In a young man affected by skin soft tissue infections complicated with myositis, the use of hyperbaric oxygen treatment as an adjuvant therapy to surgical debridement and antibiotic therapy could improve management and prognosis.

## Graphical abstract

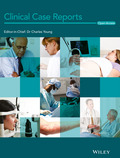

## 1 INTRODUCTION

Necrotizing soft tissue infections (NSTIs) are life‐threatening conditions firstly described by Wilson in 1952. He portrayed a rare infection characterized by bacteria‐induced necrosis of the subcutaneous tissue and fascia^1^ with a mortality rate of 6%–80%.^2^ The infection travels along planes and later can potentially involve deeper muscles with resultant myositis and myonecrosis.

The etiology is based on mono or polymicrobial infection and most frequently arises from odontogenic infections.^3^ Nowadays, the management is based on a rapid and aggressive surgical treatment with removal of the necrotic tissue along with broad‐spectrum antibiotic therapy. Some facilities have also included hyperbaric oxygen therapy (HBOT).^4^ The Italian Society of Undersea and Hyperbaric Medicine (SIMSI) has withheld the Italian authority on the indications for HBOT. In the 2nd edition of their guidelines, HBOT was also recommended as adjuvant therapy in NSTIs.^5^ Despite this, HBOT is not usually used as standard care in these patients for several reasons: Not all hospitals are equipped with a hyperbaric medicine service and a high percentage of doctors do not know the usefulness of HBOT and its possible use in these conditions. Well‐controlled, randomized, clinical trials demonstrating a statistically significant benefit of HBOT are lacking, and consequently, its use as an adjunctive therapy for NSTIs remains controversial.^6^ Besides transfer to a hospital equipped with HBOT, emergency surgical intervention should never be delayed.

We report the case of a young patient affected by NSTIs resulting from a dental infection and complicated by necrotizing myositis of the right pectoral muscle treated with standard of care plus HBOT as adjuvant therapy.

## 2 CASE REPORT

**FIGURE 1:**
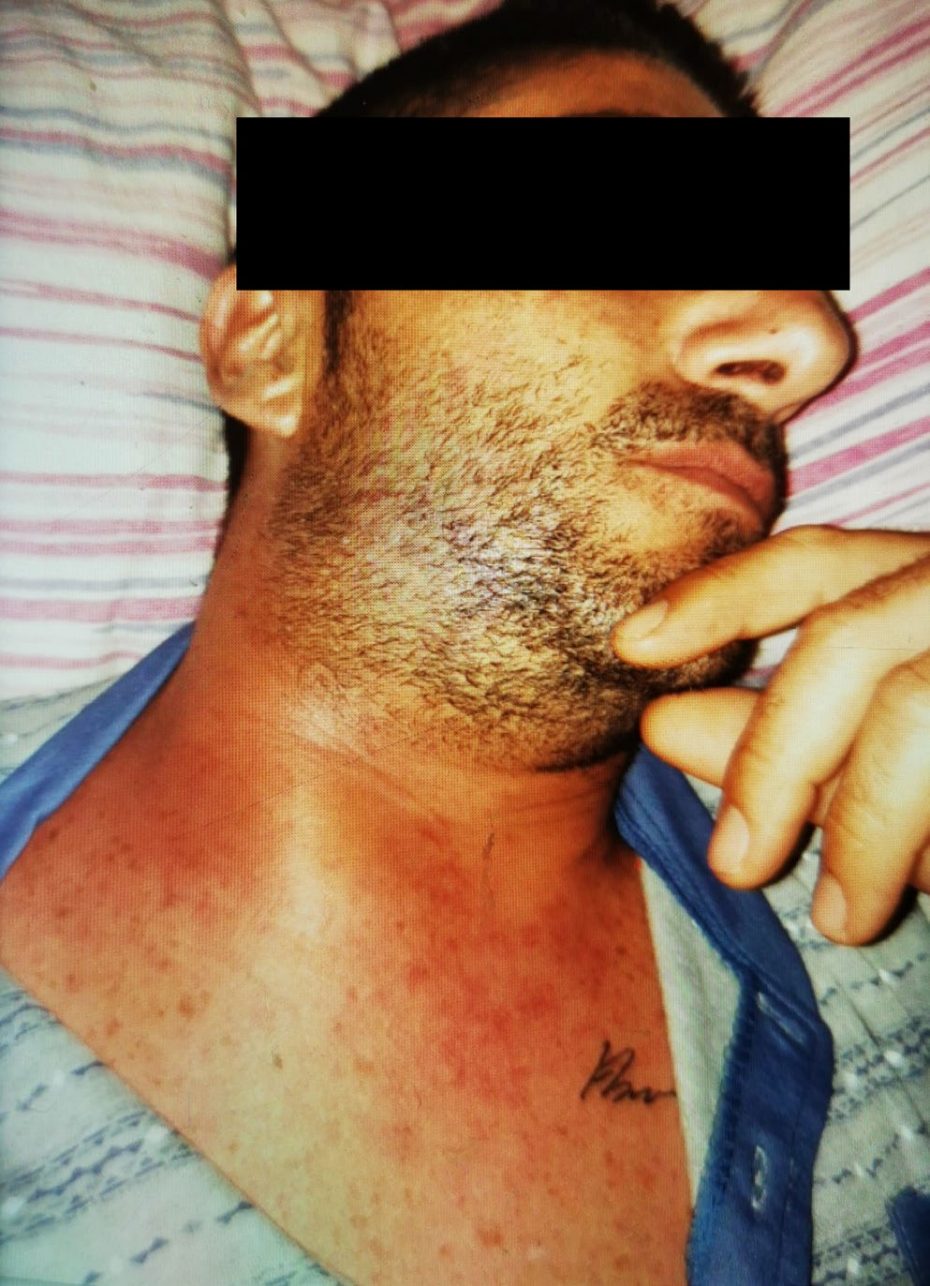
Neck of the patient before surgery: it was swollen, erythematous and tender

**FIGURE 2:**
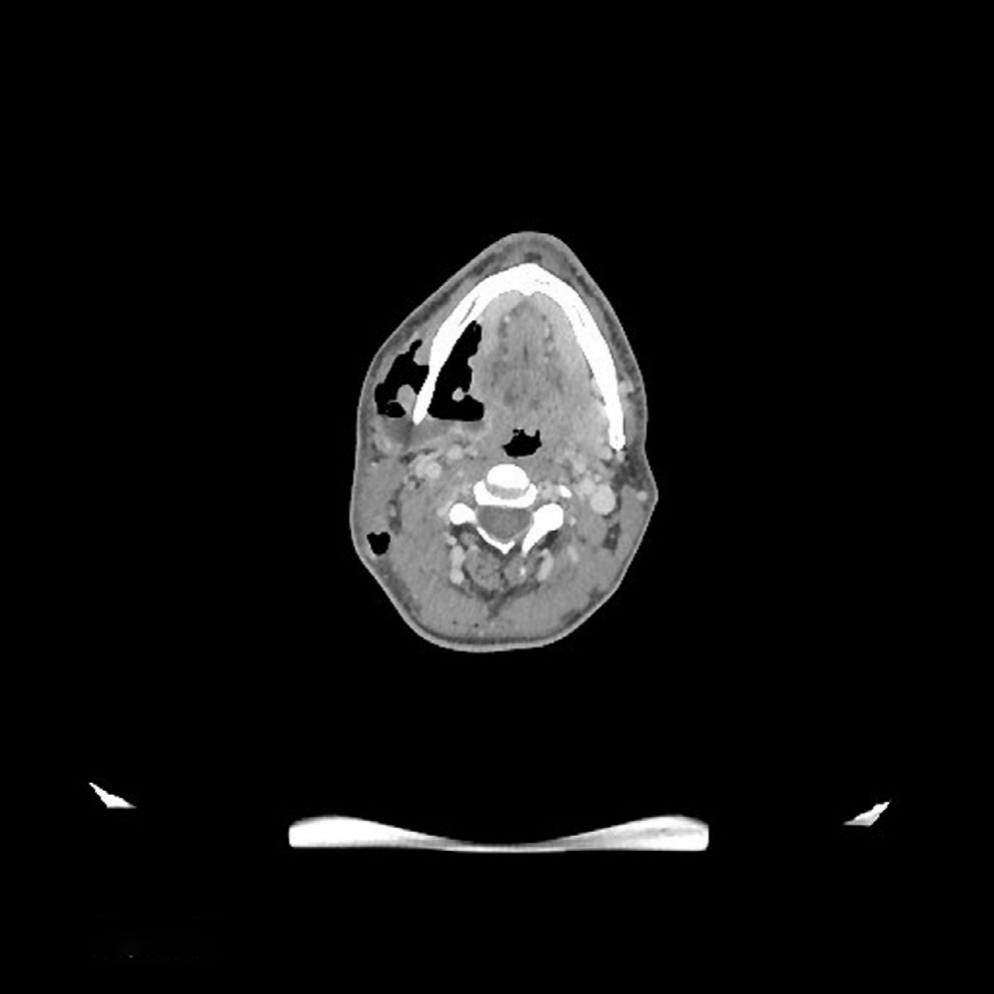
Pre‐operative CT scan of the patient's neck

**FIGURE 3:**
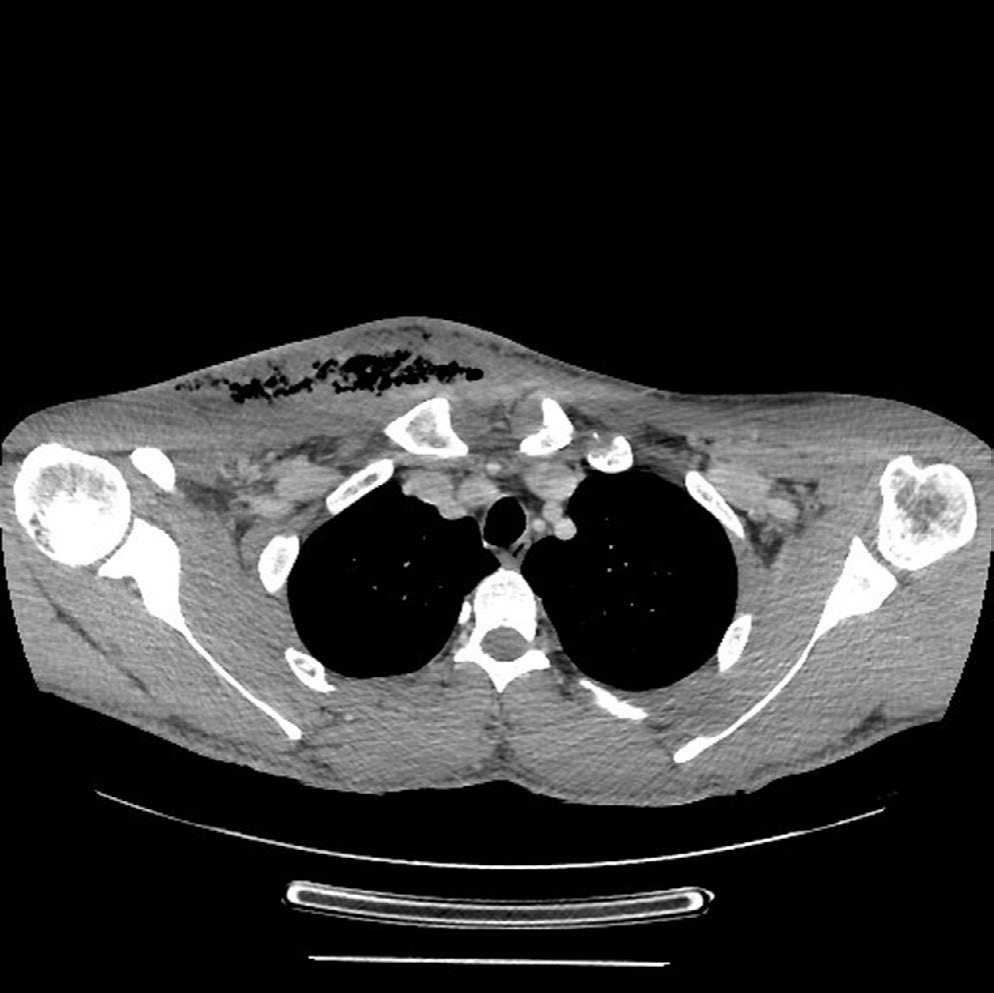
Pre‐operative CT scan of the patient's upper thorax

A 37‐year‐old man was admitted to our maxillo facial surgery department with a two‐week history of dental pain on treatment with antibiotic therapy at home. During this time, after extraction of the right lower third molar, there was progressive swelling of the submandibular and latero‐cervical region showing tense and painful skin and progressive involvement of the right upper thoracic region. The patient did not report any pathologies in his past medical history. On admission, he was conscious and cooperating, and his body temperature was 37.5°C, blood pressure 110/60 mmHg, heart rate 94 bpm, and oxygen saturation on room air 98% with no signs of respiratory exertion. Physical examination revealed swelling of the latero‐cervical region with involvement of the right pectoral region where the skin appeared tense, burning, and painful on palpation (Figure 1). The examination of the oral cavity revealed poor oral hygiene with inflammation of the gingival mucosa and leak of purulent material in the gingival fornix. A computed tomography (CT) scan of the head, neck and thorax with contrast was performed and revealed locules of gas and ill‐defined low attenuation in the cervical area and in the thigh chest muscle, highly suspicious for a gas‐forming infection (Figures 2 and 3).

Routine laboratory reports as follow: white blood cell count 15.230 mm^3^, Hb 14.5 gr/dL, CPK 414 U/L, CRP 150 mg/L, PCT 0.94 ng/mL, sodium 139 mEq/L, and potassium 4.1 mEq/L. The day after his hospital admission, the pain and swelling increased and he developed dysesthesia on the right upper thoracic zone. A compartment syndrome secondary to infection was diagnosed; thus, the patient underwent urgent extensive surgical debridement of the right pectoral area. The operating field showed interstitial “dishwater‐like” fluid and discolored poor contractile muscles with pockets of necrosis and pus. The tissues were irrigated with 10 L of saline and empiric antibiotic therapy was started with IV piperacilline‐tazobactam 4.5 gr ev/4 per day and IV daptomycin 8 mg/kg per day. The antibiotic therapy was continued for 10 days at the same dose. It was decided to quickly ensure broad antibacterial coverage for gram‐positive, gram‐negative, aerobic, and anaerobic microorganisms. The choice of these drugs was also driven by our experience in a previous study concerning SSTIs.^7^ Blood cultures were performed pre‐operatively with a sterile peripheral vein technique, and during surgery, cultures were taken from the surgical site. After surgery, the patient was extubated, and the next day, he started HBOT as adjunctive therapy. HBOT treatment consisted of two dives with 100% oxygen in a pressure chamber 2.8 atmospheres absolute (283.71 kPa) within the first two days, and then daily for a total of 16 sessions. The total time in 100% oxygen via mask for each treatment was 60 min, with three phases in room air, each lasting 5 min. The day after surgery, an increase in CPK values (660 U/L) was observed. Subsequently, a progressive reduction in CPK values was noted, which returned to values of less than 100 U/L on day eight. Similarly, we observed a progressive reduction in CRP, white blood cell, and PCT values (<20 U/L, 10,000 mm^3^ and 0.10 ng/mL, respectively). The Hb value remained substantially stable during all phases of treatment, and no blood transfusion was needed.

**FIGURE 4:**
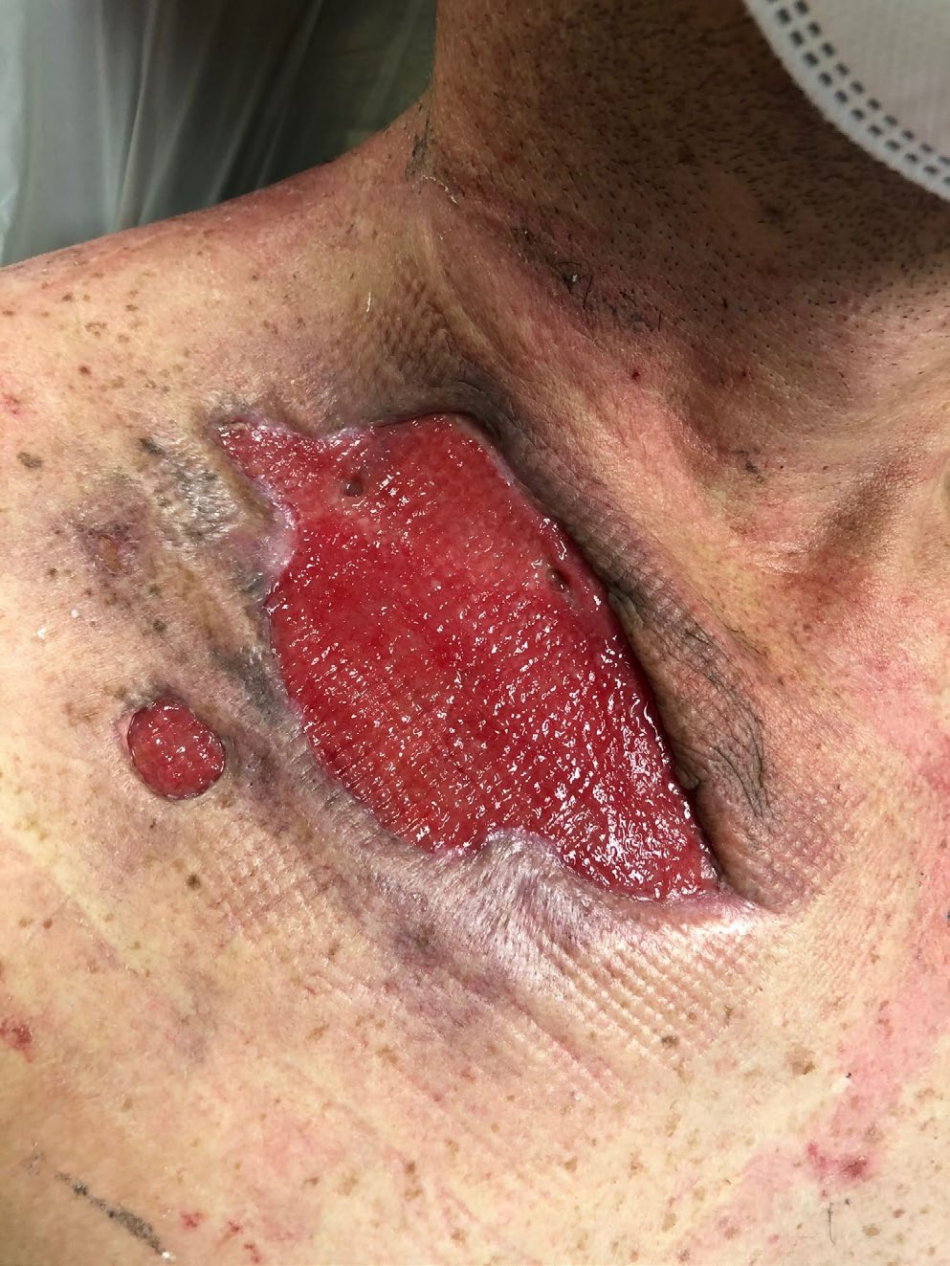
Post‐operative patient's wound, “restitutio in itinere”

**FIGURE 5:**
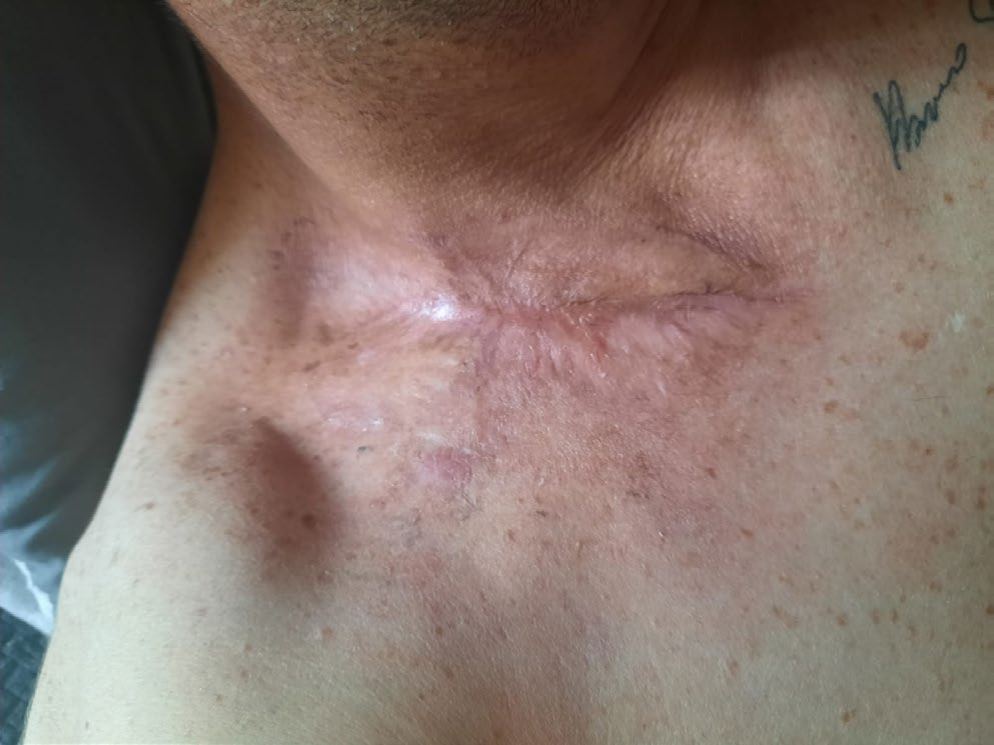
Post‐operative patient's wound: “restitution ad integrum”

Clinical improvement was observed three days after HBOT, and three weeks after admission, the patient was discharged home in good health (Figure 4). A check up three months after hospital discharge showed a complete recovery of the health status with restitutio ad integrum of the involved anatomical areas (Figure 5).

## 3 DISCUSSION

NSTIs are the heterogeneous group of infections characterized by a rapidly progressive clinical course leading to connective tissue necrosis. The initial event in the onset of NSTIs is the introduction of bacteria into the soft tissue, and this can happen as a complication of surgical procedures or medical conditions. Moreover, it may also be idiopathic, without a defined portal of entry, as in scrotal or penile necrotizing fasciitis. The prevalence is higher in males and in patients aged 45 to 64 years. Physical findings may not be commensurate with the degree of patient discomfort. Early in the course of the disease, the patient may look misleadingly well; unfortunately, this may interfere with early detection, which is the cornerstone to a favorable outcome. As reported by our previous work,^7^ the sites from which NSTIs may arise in the cervical district are represented in order of frequency by: odontogenic infection, peritonsillar abscess, and sinusitis.

According to the depth of tissue infection and necrosis, NSTIs can be classified into three forms which involve the dermis and subcutaneous tissue for necrotizing cellulitis, the fascia for necrotizing fasciitis, and the muscle layer with the intact overlying skin for necrotizing myositis.^8^

The Cervical fascia is the fibrous tissue that divide the structures of the neck and is divided into superficial and deep layers. The superficial layer of the deep cervical fascia envelops parotid and submandibular glands, and it is the external border of the odontogenic infections of the neck.^9^ Most of these infections originate from the second or third mandibular teeth as a result of infection or extraction. The roots of the second and third molars can extend up to the submandibular space and through this path the infections of this site can spread through the cervical fascia to the lateral pharyngeal space, involve the retropharyngeal space and track into the anterior or to the posterior mediastinal region with the clinical picture of mediastinitis.^10^ The infection can be monomicrobial or polymicrobial. The most frequently isolated organisms in NSTIs include viridans group streptococci, S. aureus, peptostreptococci, Bacteroides spp., Prevotella spp., and Fusobacterium.^11^ Antibiotic therapy must be started early, must be empirical and wide spectrum in the early stages, and subsequently tailored on the basis of the results obtained from cultural tests performed on biological materials. Furthermore, with regard to the choice of the drugs used, other variables should be carefully considered, such as the risk factors for multidrug‐resistant microorganisms. Finally, a further key point consists in the rapid antibiotic stewardship which must be based on clinical evidence and bio‐humoral markers. Serum PCT has been proposed as a biomarker capable of guiding the response to antibiotic therapy, allowing a rapid de‐escalation. Normal serum PCT in a healthy individual is <0.05 ng/mL. Patients with PCT values of <0.5 ng/mL are unlikely to have sepsis. However, this concentration does not exclude infection, because localized infections such as cervical NSTIs without systemic signs are still possible.^12^ In these cases, it is evident how suspecting the infectious process plays a crucial role in the diagnosis of NTSIs. The presence of low‐PCT values should be considered as an element in the puzzle that should support the clinical suspicion. Regrettably, antibiotic therapy is frequently started on an empirical basis without targeted culture samples and subsequently followed superficially.

Cervical NSTIs are uncommonly complicated by the involvement of the muscles, but when this happens, as in our case, necrotizing myositis occurs. Necrotizing myositis is a surgical emergency. It may present without skin changes; thus, it is under appreciated and diagnosis is often delayed.^13^

Clearly, when making the diagnosis of NSTIs the goal is to do it as early as possible, in order to start appropriate treatments and avoid rapid spreading of the infection and the onset of sepsis.

Several pathological conditions can affect muscle districts and differential diagnosis can be difficult. Indeed, electrolyte alterations, endocrinological disorders, a history of chronic steroid use, autoimmune disorders (such as scleroderma, systemic lupus erythematosus, myasthenia gravis, mixed connective tissue disorders and others), fibromyalgia, or rheumatic polymyalgia, should all be considered as differential diagnoses. Finally, drugs can be an important cause of myopathies, so all the drugs causing myopathy should be kept in mind, such as statins, alcohol derivates, for example, ethanol, antimetabolites (vincristine), azathioprine, chloroquine/primaquine, and anti‐fungal agents.

Our patient had no history of previous illnesses or medication intake. Moreover, poor oral hygiene, the presence of purulent material from the oral cavity and the temporal link between the dental extraction and the onset of muscular involvement made our diagnoses easier.

In our patient, the entry route was through dental infection and this is in accordance with a literature review published by Pellis et al,^14^ which highlighted how the teeth are the main (65%) responsible cause of NSTIs of the cervical region. In our case, the infection luckily did not involve the deep cervical fascial planes, but spreader along the superficial planes and affected the subcutaneous skin and muscles of the right upper thoracic region, as evidenced by the images.

HBOT has been proposed as an adjunctive therapy for patients with NSTIs.^15^ HBOT involves the inhalation of 100% oxygen at a pressure above 101.3 kPa (one atmosphere absolute = ATA). HBOT is based on several physiologic principles of how gases respond under pressure and more specifically of how oxygen responds under pressure. The increase in oxygen concentration in solution, based on its solubility under pressure, increases the diffusion gradient for its delivery deeper into tissues, which is the premise of HBOT. Because the Hb is 97% saturated in the normal body at sea level, limited improvement in oxygen delivery to tissues can be achieved by increasing Hb saturation. However, the concentration of dissolved oxygen in the plasma can be influenced greatly by HBOT. Based on Henry's Law, increased pressure will cause more gas to go into solution, and therefore,more oxygen will be transported in the plasma. Increasing the concentration of a gas within a fluid increases its partial pressure within the fluid. The increased partial pressure increases the driving force for diffusion and thereby increases its diffusion distance as defined by Fick's Law. Additionally, it is the oxygen dissolved in plasma that is most bio‐available to the tissues. By increasing the PaO2 in arterial blood, more oxygen can be delivered deeper into the tissues. Increasing the pressure from 1 ATA to 2–2.5 ATA, which is the typically working pressure with HBOT, the oxygen dissolved in plasma increases approximately 3 fold if the patient is breathing in room air. If the inhaled oxygen concentration is increased to 100% under pressure the oxygen plasma concentration increases by almost 17‐fold. In theory, with 100% oxygen at 2.5 ATA, enough oxygen can be dissolved in plasma to meet the normal requirements of the body at rest without the need for Hb. The increase in dissolved oxygen generated by HBOT has several physiologic effects that can alter tissue responses to disease and injury. The precise protocol for NSTIs varies among centers but usually consist of two or three sessions of 60–120 min at 202.6–303.9 kPa within the first 24 h. This increased oxygen availability is responsible for multiple effects as antimicrobial activity on both aerobic and anaerobic bacteria thereby demonstrating the bactericidal and bacteriostatic effects due to enhanced mobility and bacteriophagic activity of leukocytes. The killing of bacteria within leukocytes is usually divided into oxidative and non‐oxidative pathways. Oxidative killing through production of reactive oxygen species (ROS) is responsible for eliminating bacterial species that commonly infest wounds, while non‐oxidative mechanisms are responsible for less virulent bacteria that infest wounds only in the immune suppressed patient.^16^ Killing of certain bacteria by neutrophils is an energy‐ and oxygen‐dependent process that requires glucose for energy production and atmospheric oxygen for production of bactericidal oxygen‐free radical intermediates and hydrogen peroxide.^17^ Neutrophil bacterial killing activity occurs through an oxygen‐dependent respiratory burst, where neutrophils convert oxygen to superoxide.^18^ One of the main mechanisms of efficient phagocyte bacteria killing requires a local pO2 of 30 mmHg or greater.^19^ Combining early antibiotic administration and hyperoxia results in more efficient bacterial clearance than delayed antibiotics or oxygen.^20^ Increased inspired oxygen decreases the spread of infectious necrosis and increases bacterial clearance.^21^ In addition, while biofilm formation in NSTIs protects bacteria using anaerobic metabolism from antibiotics in a hypoxic environment, HBOT may restore the susceptibility to antibiotics by inducing aerobic metabolism. This has been demonstrated in Pseudomonas aeruginosa and Staphylococcus aureus biofilm models. Moreover, several recent studies have further proven the synergistic effect that HBOT can exert with antibiotic therapy in the treatment of infectious processes involving skin and soft tissues.^22^, ^23^ Finally, HBOT has shown to induce substantial effects on the expression of immune modulatory cytokines by decreasing pro‐inflammatory cytokines such as IL‐1, IL‐6, and TNF‐alfa and elevating the anti‐inflammatory cytokine IL‐10.^24^

It is interesting to report other effects that HBOT is able to exert on wounds as it encourages collagen production by fibroblast, which leads to the growth of granulation tissue, an essential step in wound healing.^25^

Moreover, the increase in oxygen tension, which persists for several hours after therapy, is responsible for angiogenic properties of HBOT.^26^ Hyperbaric oxygen causes reduced platelet aggregation, improved tissue microcirculation, and shortens metabolic disorders. These properties along with increased dissolved oxygen in plasma lead to better oxygenation of hypoxic tissue, where red blood cells cannot reach.^27^

Although numerous studies have clearly outlined the benefits that HBOT can have in the treatment of these diseases, the findings in term of outcome are controversial. A study analyzed clinical data retrospectively for 78 patients with NSTIs: 30 patients were treated with standard of care (surgery plus antibiotics), and 48 received adjunctive HBOT. The mortality rate for HBOT group was lower though not significantly different than that observed for the non‐HBOT group.^28^ On the contrary, a single‐center, retrospective, case‐controlled study assessed the effect of HBOT in reducing mortality in patients with NSTIs. This study involved a total of three hundred and forty one patients. The mortality rate was 12% and 24.3% in patients treated with HBOT and not treated respectively. The authors concluded that mortality was linked to illness severity at presentation. However, when adjusted for severity score and need for intensive care management, HBOT was associated with a significant reduction in mortality.^29^

The hyperbaric treatment of our patient was conducted on the basis of the SIMSI guidelines^5^: In the first three days, we adopted the maximum oxygen dosage at 2.8 ATA (284 kPa), twice a day. From day four, treatments are performed daily with sessions at 2.5 ATA (253 kPa). The duration of each session was 80 min, and the treatment was extended until the infection was considered to be overcome, condition also confirmed by the return of infection biomarker within normal limits. Our patient showed excellent compliance with the method throughout the treatment.

Absolute contraindications to HBOT are represented by non‐drained pneumothorax, PaO2/FiO2 ratio <200 assessed by blood gas analysis, seizures, claustrophobia, and psychiatric problems. HBOT carries a limited number of risk complicating the therapeutic process for patients with NSTIs. When examining the complications of HBOT, there are two categories: pressure related and oxygen related. The first category is barotrauma, which can affect any closed, air filled cavity. Middle ear barotrauma occurs in about 2% of awake patients^30^ but is avoided in unconscious patients by the use of trans‐tympanic ventilation tubes. Pulmonary barotrauma may rarely happen during decompression in patients with airway obstruction. The second category can further be subdivided in three different complications: pulmonary, neurologic, and ophthalmologic. Pulmonary toxicity requires prolonged exposure to hyperbaric doses and is not a practical problem. The incidence of oxygen seizures is approximately 0.01% of treatments with no evidence of long‐term sequelae.^31^ The most common ocular complication of hyperbaric oxygen therapy is myopic change. It is thought to be fully reversible after the cessation of hyperbaric oxygen therapy, with the vision rapidly improving after 3 to 6 weeks. However, fully returning to baseline can take as long as one year. Confinement anxiety is more an effect of the physical space of the chamber and not a true complication.

## 4 CONCLUSION

NSTIs are rare but potentially fatal diseases. A high index of suspicion is required for early diagnosis because signs and symptoms in the early stages of infection are poor indicators of illness severity. Our case report suggests that HBOT may be a clinically useful adjunctive treatment. In fact, most recommendations have been made with a low level of evidence because of the lack of randomized controlled studies available in this field. Therefore, it should be kept in mind that HBOT must not replace the combination of early aggressive surgical debridement and wide‐spectrum antibiotic therapy, but rather could be considered as a potential adjuvant treatment. Prompt HBOT could be useful for preventing mortal complications and for reducing costs, associated with the management of these patients, through a shorter length of stay and a reduction in relapses. As this technology becomes more available to clinical practice, HBOT should be considered as a therapeutic option.

## CONFLICT OF INTEREST

No Author has conflict of interest statement.

## AUTHOR CONTRIBUTIONS

Andrea N. Cracchiolo and Daniela Maria Palma have been involved in drafting the manuscript or revising it critically for important intellectual content; Marco Palmeri, Diego Tantillo, Rosalia Lo Bue, Andrea Braconi, Claudio Caramanna, Luigi Solazzo and Fabio Genco have made substantial contributions to conception and design, or acquisition of data, or analysis and interpretation of data; Paola Mirto gave final approval of the version to be published. Each author participated sufficiently in the work to take public responsibility for appropriate portions of the content; and agreed to be accountable for all aspects of the work in ensuring that questions related to the accuracy or integrity of any part of the work are appropriately investigated and resolved.

## ETHICAL APPROVAL

All procedures followed were in accordance with the ethical standards of the responsible committee on human experimentation (institutional and national) and with the Helsinki Declaration of 1975, as revised in 2000.

## Data Availability

The data that support the findings of this study are available on request from the corresponding author. The data are not publicly available due to privacy or ethical restrictions.
